# From Suspected Encephalitis to Catatonia and First-Episode Psychosis in a Neuro-Vulnerable Refugee Adolescent

**DOI:** 10.1192/bjo.2026.11793

**Published:** 2026-06-30

**Authors:** Chris Popoola, James McClung, Praveen Kumar

**Affiliations:** 1 University of Dundee, Dundee, United Kingdom; 2 Imperial College London, London, United Kingdom; 3 NHS Grampian, Aberdeen, United Kingdom

## Abstract

**Aims:**

Paediatric encephalitis is a medical emergency, and empirical treatment is used, given risk of neurological sequelae and death. Catatonia and first-episode psychosis can mimic encephalitis clinically, especially in medically complex children. This case describes an adolescent initially treated for suspected encephalitis.

**Methods:**

A neuro-vulnerable 13-year-old refugee girl from Kenya presented to hospital with acute behavioural and mental state change with functional decline preceded by flu infection and major psychosocial stress due to deportation threat. Her medical history includes bilateral glaucoma, cataracts, basal ganglia calcifications, and left temporal arachnoid cyst. The initial working diagnosis was encephalitis and first-episode psychosis. IV methylprednisolone, cefotaxime, aciclovir, and immunoglobulin were commenced, followed by an oral prednisolone taper. Amlodipine 5mg once daily achieved good blood pressure control. Negative results from medical work-up (including neuro-inflammatory process and neuronal antibodies) led to immunotherapy being discontinued. During this time, catatonia developed (Bush-Francis Score of 14), 6kg was lost due to reduced intake, and psychotic symptoms continued. A lorazepam challenge showed substantial improvement in catatonia, engagement, and eating. Aripiprazole showed progressive reduction in psychotic symptoms. The patient was transferred to an adolescent mental health unit. Formulation: catatonia and psychosis in a highly neuro-vulnerable adolescent, triggered by acute psychosocial stress around asylum rejection and fear of return to an abusive environment. Lorazepam and aripiprazole were continued.

**Results:**

Since encephalitis is such a serious condition and takes several days of waiting on tests for diagnosis confirmation, treatment is initiated once encephalitis is suspected. Although it presents non-specifically, suggestive signs include fever, personality change, and reduced consciousness, which fit the case presentation. Subsequent treatment of IV steroids and anti-virals is in line with past literature although use of steroids (often initiated to control raised intracranial pressure) is under further research. Once encephalitis was ruled out, appropriate treatment for psychosis and catatonia was commenced. Due to complex medical history, a genetics test was ordered and results were suggestive of oculodentodigital syndrome (GJA1-related). Two variants of uncertain significance have been found in GJA1 and parental samples are being analysed.

**Conclusion:**

Catatonia and first-episode psychosis can mimic encephalitis. Clinicians should treat suspected encephalitis urgently and maintain a low threshold to re-evaluate diagnosis, integrating psychosocial context (in this case, refugee status) and catatonic signs to guide psychiatric intervention. Constellations of symptoms can be indicative of syndrome diagnosis. Genetic testing should be initiated as required.

